# Pylephlebitis – an uncommon challenging entity

**DOI:** 10.4322/acr.2024.480

**Published:** 2024-03-15

**Authors:** Vitorino Modesto dos Santos, Lister Arruda Modesto dos Santos

**Affiliations:** 1 Universidade Católica, Hospital das Forças Armadas, Departamento de Medicina, Brasília, DF, Brasil; 2 Hospital Francisco Morato de Oliveira, Serviço de Cirurgia Geral, São Paulo, SP, Brasil

Dear Editor,

Pylephlebitis, or thrombophlebitis of the portal vein system, is an infrequent condition mainly associated with Gram-negative bacteria,^1-5^ which has been related to the origin of liver abscesses since almost three centuries ago;^1,4^ with an estimated prevalence of 7%,^2^ and an incidence that ranges from 0.3 to 3 cases per 100,000 patients per year.^1,4^ Pancreatitis, diverticulitis, peritonitis, appendicitis, and cholecystitis are the major associated entities. *Bacteroides fragilis*, *Escherichia coli*, *Klebsiella spp*, *K. pneumoniae*, *Proteus mirabilis*, *Enterobacter spp*, *Staphylococcus spp*, *Streptococcus spp*, *S. constellatus*, *Fusobacterium necrophorum*, *Bacteroides ovatus*, *Clostridium*, and *Enterococcus* are the related etiological agents.^1-5^ Classical pathological features of pylephlebitis include polymorphonuclear infiltrates, venulitis with fibrinoid necrosis, and suppurative involvement of hepatic parenchyma.^1,4^ The endothelial activation in the portal vein and tributaries by the infection can cause local thrombosis, which may evolve into portal hypertension and cavernomatous transformation, while septic emboli give origin to abscesses in the lungs and central nervous system.^1^ Therefore, immediate antibiotic therapy constitutes the major therapeutic tool, either mono or combined therapy, while anticoagulation varies from case to case. ^1-5^ Due to nonspecific clinical manifestations, the imaging workup is mandatory for the early diagnosis of the challenging entity with an ominous course when it is not promptly treated.^1-5^

We read the illustrative case study by Martins et al.^4^ of a 56-year-old diabetic male who died due to septic shock on Day 1 of admission in an intensive care unit. He had unspecific manifestations of acute abdomen, and the autopsy established the final diagnoses of calculous cholecystitis, pylephlebitis, and liver abscesses. ^4^ The authors commented on the delayed diagnosis and treatment favoring increased morbidity and mortality and that cholecystitis is reported in nearly 7% of pylephlebitis.^4^

This letter aims to highlight the important role of the manuscript recently published in this Journal, calling the attention of primary healthcare workers to a potentially ominous, very challenging cause of acute abdominal manifestations. ^4^ Artese et al.,^1^ described an 11-year-old male patient who presented pylephlebitis, liver abscesses, septic shock by *E. coli*, and evolved with ascites and right pleural effusion.^1^ He underwent piperacillin-tazobactam and anticoagulation with enoxaparin; the images of control revealed thrombosis, cavernomatous transformation, and portal hypertension.^1^ After a long-standing follow-up, he maintained normal liver function and nutritional status; the authors stressed the importance of early diagnosis in the prevention of irreversible complications.^1^ Faye et al.^2^ reported a 43-year-old female presenting fever, abdominal pain, vomiting, leukocytosis, high C-reactive protein, and inflamed distended gallbladder with lithiasis; besides evidence of pylephlebitis at the portal trunk and the right portal branch. After the hospital admission, she underwent ceftriaxone and metronidazole with a good response and was discharged on day 7, already with a scheduled cholecystectomy.^2^ The authors highlighted the challenging clinical manifestations and the useful role of tomography images in confirming the diagnosis and clearly identifying associated etiological factors.^2^ Hapshy et al.^3^ described a 42-year-old man with fever, acute abdominal pain, jaundice, mental changes, tachycardia, and arterial hypotension, besides leukocytosis. However, the abdominal and chest imaging studies did not detect the infection site; blood cultures revealed the presence of *Fusobacterium necrophorum* and *Bacteroides ovatus*.^3^ Noor et al.^5^ reported a 73-year-old man who presented lower abdominal pain, nausea, and chills manifested two weeks after the laparoscopic cholecystectomy for cholelithiasis. There was leukocytosis with neutrophilia, positive blood culture for *S. constellatus*, and imaging studies revealed the left and right main portal veins occluded by thrombus; therefore, he underwent enoxaparin and ceftriaxone.^5^ The authors emphasized the rarity of the pylephlebitis post laparoscopic cholecystectomy, the role of imaging studies to confirm this challenging diagnosis, the need for antibiotic therapy guided by cultures, and anticoagulation to prevent chronic portal hypertension.^5^

Considering that case studies represent most of the current published manuscripts related to pylephlebitis,^4^ the authors strongly believe that wide acknowledgement of the respective data can contribute to reducing the number of misdiagnoses, besides increasing the suspicion index of non-specialists, which favors early diagnoses.

## References

[1] Artese D, Romero JI, Álvarez NP (2024). Pylephlebitis in pediatrics: a diagnostic challenge. Arch Argent Pediatr.

[2] Faye I, Ndong A, Diallo AC (2023). Pylephlebitis complicating acute calculous cholecystitis: a case report. Radiol Case Rep.

[3] Hapshy V, Imburgio S, Sanekommu H (2024). Pylephlebitis-induced acute liver failure: a case report and review of literature. World J Hepatol.

[4] Martins WD, Santana JPB, Barros MF, Duarte AN (2024). Pylephlebitis. Autops Case Rep.

[5] Noor J, Chaudhry A, Batool S (2023). Pylephlebitis complicated by bacteremia: a rare complication following laparoscopic cholecystectomy. Cureus.

